# Prognostic significance and identification of basement membrane-associated lncRNA in bladder cancer

**DOI:** 10.3389/fonc.2022.994703

**Published:** 2022-10-10

**Authors:** Lixiang Feng, Jun Yang, Wei Zhang, Xiong Wang, Lili Li, Min Peng, Pengcheng Luo

**Affiliations:** ^1^Department of Urology, Wuhan Third Hospital, School of Medicine, Wuhan University of Science and Technology, Wuhan, China; ^2^Department of Urology, Wuhan Third Hospital, Wuhan, China; ^3^Department of Pharmacy, Wuhan Third Hospital, Wuhan, China; ^4^Central Laboratory, Renmin Hospital of Wuhan University, Wuhan, China; ^5^Department of Oncology, Renmin Hospital of Wuhan University, Wuhan, China

**Keywords:** bladder cancer, basement membrane, prognosis, lncRNAs, biomarkers

## Abstract

Based on the importance of basement membrane (BM) in cancer invasion and metastasis, we constructed a BM-associated lncRNA risk model to group bladder cancer (BCa) patients. Transcriptional and clinical data of BCa patients were downloaded from The Cancer Genome Atlas (TCGA), and the expressed genes of BM-related proteins were obtained from the BM-BASE database. We download the GSE133624 chip data from the GEO database as an external validation dataset. We screened for statistically different BM genes between tumors and adjacent normal tissues. Co-expression analysis of lncRNAs and differentially expressed BM genes was performed to identify BM-related lncRNAs. Then, differentially expressed BM-related lncRNAs (DEBMlncRNAs) between tumor and normal tissues were identified. Univariate/multivariate Cox regression analysis was performed to select lncRNAs for risk assessment. LASSO analysis was performed to build a prognostic model. We constructed a model containing 8 DEBMlncRNAs (AC004034.1, AL662797.1, NR2F1-AS1, SETBP1-DT, AC011503.2, AC093010.2, LINC00649 and LINC02321). The prognostic risk model accurately predicted the prognosis of BCa patients and revealed that tumor aggressiveness and distant metastasis were associated with higher risk scores. In this model, we constructed a nomogram to assist clinical decision-making based on clinicopathological characteristics such as age, T, and N. The model also showed good predictive power for the tumor microenvironment and mutational burden. We validated the expression of eight lncRNAs using the dataset GSE133624 and two human bladder cancer cell lines (5637, BIU-87) and examined the expression and cellular localization of LINC00649 and AC011503.2 using a human bladder cancer tissue chip. We found that knockdown of LINC00649 expression in 5637 cells promoted the proliferation of 5637 cells.Our eight DEBMlncRNA risk models provide new insights into predicting prognosis, tumor invasion, and metastasis in BCa patients.

## Introduction

Bladder cancer (BCa), a tumor that grows in the bladder mucosa, ranks the fourth most aggressive tumor among male-related cancers (1). In today’s clinical treatment, the cystoscopic biopsy is regarded as the gold standard for BCa detection (2). BCa is divided into two categories according to whether there is tumor infiltration in the bladder muscle: non-muscle-invasive type and muscle-invasive type. Nearly 70% of all newly diagnosed BCa patients are non-muscle invasive bladder cancer (NMIBC) (3). Tumor resection is the most common treatment method for NMIBC, followed by intravesical Bacillus Calmette-Guerin (BCG) immunotherapy or intravesical chemotherapy (4). The 5-year survival rate of NMIBC is approximately 90%, and the risk of postoperative recurrence is 50% to 70% (5). Nearly 25% of NMIBC patients will eventually break through the basement membrane barrier and develop into muscle-invasive bladder cancer (MIBC), which may be related to drug resistance (6). Although the current combination of surgery, radiotherapy, chemotherapy and targeted therapy has extended the overall survival of patients to a certain extent, the overall patient recurrence and mortality rates of BCa are still high (4). Due to the properties of BCa, personalized medicine is currently the most used approach to benefit patients and treatment outcomes. However, developing better-personalized medicine requires more validated biomarkers, such as early diagnosis and prognostic indicators, which can help doctors identify patients who need early aggressive treatment earlier and predict patients who respond to the targeted drug (7). In recent years, immunotherapy with immune checkpoint inhibitors has been gradually used for treatment and has achieved certain curative effects. This approach includes anti-PD-L1 therapies, atezolizumab, avelumab, nivolumab and pembrolizumab, which are only approved in the metastatic stage (8–10). However, relatively few biomarkers are available to assess the efficacy of immunotherapy against BCa. Therefore, there is an urgent need for new and precise efficacy assessments of BCa therapy.

At present, most cancer patients die from cancer metastasis rather than the primary tumor. Almost 90% of cancer deaths are due to cancer metastasis (11). At the beginning of BCa, cancer begins as noninvasive papillary carcinoma (or carcinoma *in situ*), with 30 abnormal proliferation of cancer cells in the urothelium. The tumor can be surgically removed at this stage but has a high recurrence rate. BCa can evolve into an aggressive tumor when passing through the basement membrane (BM). Cancer cells can enter the connective tissue, get more space, nutrients and oxygen, increase in large quantities, enter the muscle layer, capillaries, and finally, cancer cells transfer (12). The BM mainly comprises a laminin and collagen network. Under normal circumstances, cells cannot pass through it. When the BM is dysregulated, it can promote the invasion and migration of cancer (13). BM is a vital tissue barrier between *in situ* and invasive carcinoma. The formation of endosomes and the degradation of extracellular matrix are the key to tumor invasion (14). Studies have shown that BM can be degraded mediated by MMP-2 and MMP-9, which are matrix metalloproteinases (MMPs) with proteolytic activity (15). lncRNAs can regulate BM degradation by affecting BM-associated proteins. BM-associated lncRNAs can not only serve as direct biomarkers to help differentiate cancers but also help clinicians monitor BM status to assess the extent of cancer progression. Therefore, developing BM-related lncRNA biomarkers is crucial for evaluating the degree of cancer invasion.

There are few studies on BM-related lncRNAs, so more research data on lncRNAs are needed to provide new references for clinical treatment. Several BM-related lncRNAs are closely associated with tumor infiltration and invasion. For example, lncRNA FOXF1-AS1 promotes the migration and invasion of osteosarcoma cells by promoting MMP-2 and MMP-9 (16). PSMA3-AS1 can upregulate laminin subunit gamma 1 (LAMC1) to promote the proliferation and migration of cholangiocarcinoma (17). BBOX1-AS1 promotes the proliferation and metastasis of oral squamous cell carcinoma and inhibits apoptosis by upregulating laminin subunit gamma 2 (LAMC2) (18).

This study aimed to explore differentially expressed BM-related lncRNAs (DEBMlncRNAs) in BCa and evaluate their prognostic significance. RNAseq, clinical, and somatic mutation data of BCa patients were downloaded through The Cancer Genome Atlas (TCGA) database. BM-related protein expression genes were obtained through the BM-BASE database. In the research, we identified eight BM-related lncRNAs to construct prognostic risk models, which could enhance prognostic prediction in BCa patients with various clinical conditions. We further analyzed differences in clinical characteristics and associated prognosis by risk-prognosis grouping, nomogram, functional enrichment analysis, tumor mutational burden analysis, immune function analysis, and immune infiltration assessment. We validated the expression of eight risk model DEBMlncRNAs in BCa cells. This model lays the foundation for studying immune mechanisms, new therapeutic targets, and clinical drugs.

## Materials and methods

### Cell culture

Two human BCa cell lines (5637 and BIU-87) and the normal human bladder epithelial cell line SV-HUC-1 were used in this study. All three cells were obtained from IMMOCELL (Xiamen, Fujian, China). Human bladder cancer Cell line 5637 and BIU-87 were cultured in RPMI-1640 medium, and SV-HUC-1 was cultured in F-12K medium, both of which were supplemented with 10% fetal bovine serum (FBS) and 1% Penicillin-Streptomycin solution. The cells were then cultured in a humidified incubator at 37°C with 5% CO_2_.

### Identification of differentially expressed BM-related lncRNAs

In total, we collected 222 BM genes from the BM-BASE database, and 79 differentially expressed BM genes were identified using the Limma package in the R project (log2|fold change (FC)| ≥ 1.0, and *p* < 0.05) (19). A total of 14,056 lncRNAs were extracted from the transcriptome data of BCa patients in the TCGA database. Furthermore, correlation analysis between 79 differentially expressed BM genes and 14056 lncRNAs was identified by Pearson correlation analysis. A total of 435 BM-related lncRNAs (BMlncRNAs) were identified in BCa (|cor| > 0.4, *p* < 0.001). Finally, 304 DEBMlncRNAs were obtained by using the Limma package (log2|FC| ≥ 1.0 and *p* < 0.05) (19).

### Construction of the prognostic signature

396 BCa samples acquired from TCGA were randomly separated into a training cohort and a test cohort in a 1:1 ratio to create a lncRNA-based signature. By univariate Cox analysis, we identified potential DEBMlncRNAs that showed great prognostic value for BCa in the training cohort (*p* < 0.05). Then, we conducted a LASSO regression analysis to remove the overfitting variables. Subsequently, a DEBMlncRNA signature was further generated using multivariate Cox regression to analyze the hazard ratios of potential lncRNAs. The risk of DEBMlncRNA signature = Σexp (DEBMlncRNAs) × β, where β is the coefficient of each candidate DEBMlncRNA from the multivariate Cox analysis.

### Evaluation and validation of the risk model

Risk scores were analyzed for each BCa patient. All patients were divided into high-risk groups (high-risk score) and low-risk groups (low-risk score) according to the median risk score. The KM survival curves were compared between the two risk groups’ overall survival, disease-specific, and progression-free survival. Time-dependent receiver operating characteristic (ROC) curve analysis of 1-year, 3-year, and 5-year survival rates and risk scores and various clinical features were performed using the “survival”, “survminer” and “timeROC” R packages (20). The expression of 8 lncRNAs in high-risk and low-risk groups was visualized with a heatmap, and the distribution of risk score and survival time for each patient was described using a scatterplot.

### Construction of the predictive nomogram

For the univariate and multivariate Cox regression analysis, a total of 396 cases with accompanying clinical data were employed. To improve the predictive potential of DEBMlncRNAs, we constructed a nomogram derived from DEBMlncRNAs and other clinicopathological characteristics for outcome forecasting in BCa patients. To validate the nomogram, calibration curves were created.

### Risk score and clinical characteristics

Prediction of prognosis by age, gender, and T stage in high-risk and low-risk groups using KM survival curves. A heatmap showed the relationship between 8 lncRNAs and clinical features in the risk model. Boxplots showed differences in clinical characteristics in risk scores.

### Functional and pathway enrichment analysis

We screened for differentially expressed genes (DEGs) between high-risk and low-risk groups of BCa patients in TCGA by using the R package Limma (19). The screening criteria were |log2FC| ≥ 1 and *P* < 0.05. Gene ontology (GO) analysis to explore DEGs related to gene functions. Gene Set Enrichment Analysis (GSEA) and Kyoto Encyclopedia of Genes and Genomes (KEGG) were used to find signaling pathways associated with DEGs.

### Analysis of tumor-infiltrating immunocyte and immune checkpoints

CIBERSORT is an immune correlation algorithm that analyzes the abundance of 22 immune cells in a sample to show the immune status of BCa samples. We used the R packages ggplot2 and ggpubr to visualize risk scores and immune checkpoint activation between patients in the low-risk group and patients in the high-risk group (20, 21).

### Estimation of tumor mutational burden

Tumor mutational burden (TMB) is a new therapeutic metric used to determine sensitivity to immunotherapy. The somatic mutation data were analyzed using the R package maftools (22). The median TMB value was used as a demarcation criterion to classify BCa patients into high TMB and low TMB groups.

### Exploration of the model in clinical treatment

After downloading data from the Genomics of Cancer Drug Sensitivity (GDSC) database, the R package pRRophetic was used to analyze the half-maximal inhibitory concentration (IC50) of each BCa patient and the drug to predict treatment response (23).

### Validation of risk model LncRNA expression with external datasets

Gene expression data of BCa and paracancerous tissues in GSE133624 were downloaded from the GEO database, and a total of 36 BCa tissues and 29 paracancerous tissues were obtained. We used the R package Limma to examine the differential expression of risk model lncRNAs in BCa and paracancerous tissues. The test criteria were |log2FC| ≥ 1 and P < 0.05 (19).

### RNA isolation and quantitative real-time polymerase chain reaction

mRNA was extracted using Trizol reagent (Invitrogen, Carlsbad, CA, USA) and was reversed to cDNA by using ReverTra Ace^®^ kit (Toyobo, Japan) according to the manufacturer’s protocol. qRT-PCR was used to detect related genes by using SYBR green PCR master mix (Servicebio, Wuhan, China). The primer sequences were listed as follow: AC004034.1, forward, 5’-CCTGTGAGACCCTGAGCAGAGG-3’ and reverse, 5’-ATGGTAGGCTAAGTCCTGTGAGTCC-3’; AL662797.1, forward, 5’-CGGATCTTGCTGATAAGGAGAGTGC-3’ and reverse, 5’-ATGTGGTACGGAAGGAGGCAGAG-3’; NR2F1-AS1, forward, 5’- CGGCACAGCAGACCTCTTAGTAATG-3’ and reverse, 5’- CAACAGATTGGCTGGAGGATGGTAG-3’; SETBP1-DT, forward, 5’- TGGCTGCTGGTTTGAGTTCCTTC-3’ and reverse, 5’- CCCAGTCTCTTTCACTCCACTTCAC-3’; AC011503.2, forward, 5’- CTTCGCCTCATACTTGCTCTGTCTC-3’ and reverse, 5’- GCATCTGCTTCTGGTGAGAGTGTC-3’; AC093010.2, forward, 5’- CCATAAGTCTCGGCACTGCTCATC-3’ and reverse, 5’- GACTTCCCAGTATGGCGTTTCTCC-3’; LINC00649, forward, 5’- AGACACTTGCGGTTCTTCCATTGAG-3’ and reverse, 5’- GGTGCCTCAGATGCTACTGGTTATG-3’; LINC02321, forward, 5’- TGGTGAGGGTTGGTGAGCAGAC-3’ and reverse, 5’- CCCAGAGGAACGCCAGGAATTAAC-3’.

### Cell transfection and CCK-8 assay

5637 cells were grown in 1640 medium (Servivebio) containing 10% fetal bovine serum (EVERY GREEN) in an incubator (37°C and 5% CO2). Small interfering RNA targeting LINC00649 (si-LINC00649), as well as negative control RNA (si-NC), were transfected into 5637 cells by Lipo 2000 (Invitrogen). The viability of 5637 cells was detected using the CCK-8 assay kit (Krbio) according to the manufacturer’s instructions. Optical density was measured by a microplate reader (BIOTEK).

### RNA FISH

BCa tissue chips (Biotechwell) were fixed with 4% formaldehyde/10% acetic acid and stored in 70% ethanol overnight. A fluorescently labelled single-stranded probe (lncRNA LINC00649: AGTTGGAAAGGTCCCGCTAGTTGA-Cy3; lncRNA AC011503.2: AGAGACAGAGCAAGTATGAGGCGAA-Cy3) was synthesized, followed by hybridization. 18S and U6 oligonucleotides were purchased from Ribo Bio (Shanghai). To increase the stability of RNA foci, RNA signal was detected with the tyramideAlexa Fluor 546 Signal Amplification Kit (Invitrogen). After labelling, fluorescence signals were detected using a microscope (BX41; Olympus).

### Statistical analysis

R version 4.1.2 was used to examine all statistical data. Kaplan-Meier survival analysis was performed to detect survival distinctions between the two risk groups. Statistical analysis was performed using flexible statistical methods and was statistically significant when the *p*-value was less than 0.05.

## Results

### Acquisition of differentially expressed BMlncRNAs

**Figure 1:**
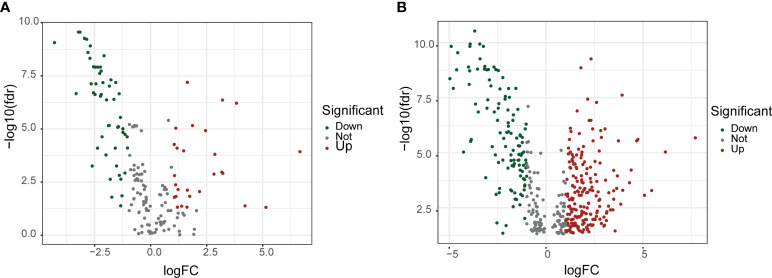
Screening for basement membrane-associated differential genes and differential LncRNAs. **(A)** Volcano plot of 79 basement membrane-associated differential genes. **(B)** Volcano plot of 304 basement membrane-associated differential LncRNAs.

From the BCa transcriptome data of TCGA, a total of 14,056 lncRNAs were extracted. From the BM-BASE database, 222 BM genes were obtained, and 79 differentially expressed BM genes were identified, comprising 27 up-regulated genes and 52 down-regulated genes (**Figure 1A**). Then, the co-expression relationship between 14,056 lncRNAs and 79 differentially expressed BM genes was analyzed. A total of 435 lncRNAs were identified as BM lncRNAs. Finally, 304 DEBMlncRNAs were identified (**Figure 1B**).

### Development of a prognostic risk model

**Figure 2:**
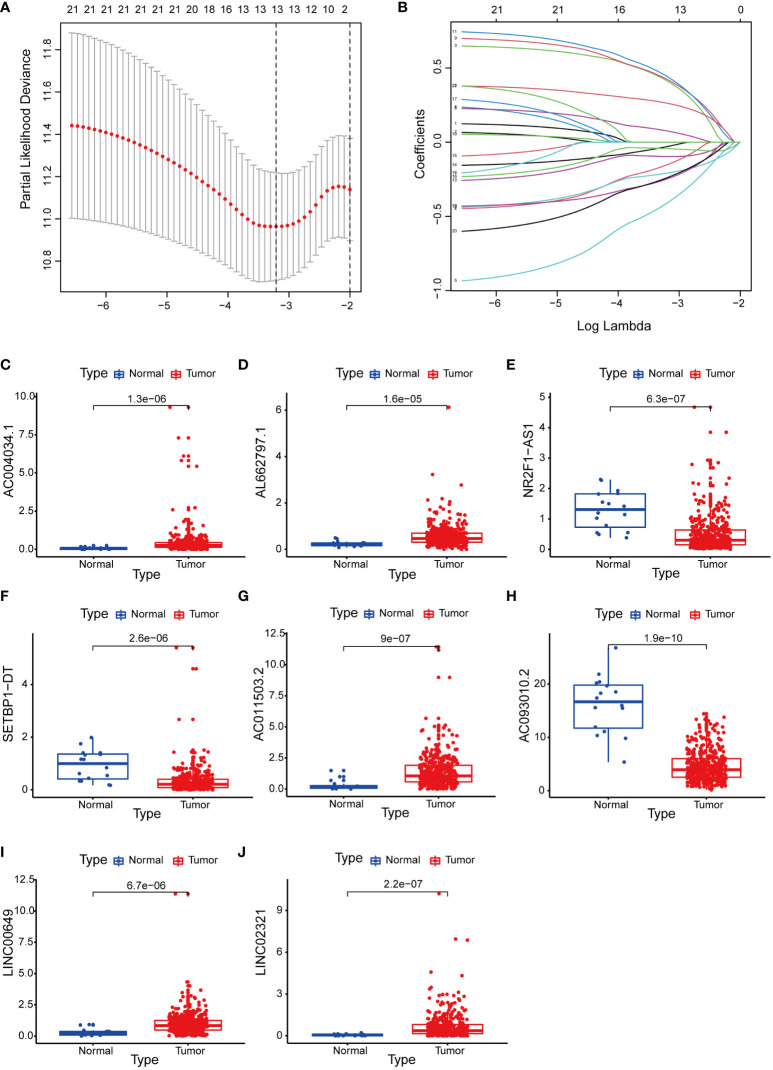
Construction of a basement membrane-associated lncRNA risk model. **(A)** The LASSO coefficient profile of 8 differential basement membrane-associated lncRNAs. **(B)** Variable cross-validation in LASSO regression. Expression of 8 lncRNAs **(C)** AC004034.1 **(D)** AL662797.1 **(E)** NR2F1-AS1 **(F)** SETBP1-DT **(G)** AC011503.2 **(H)** AC093010.2 **(I)** LINC00649 **(J)** LINC02321 in bladder cancer and adjacent paracancerous tissues from the TCGA database.

**Figure 3:**
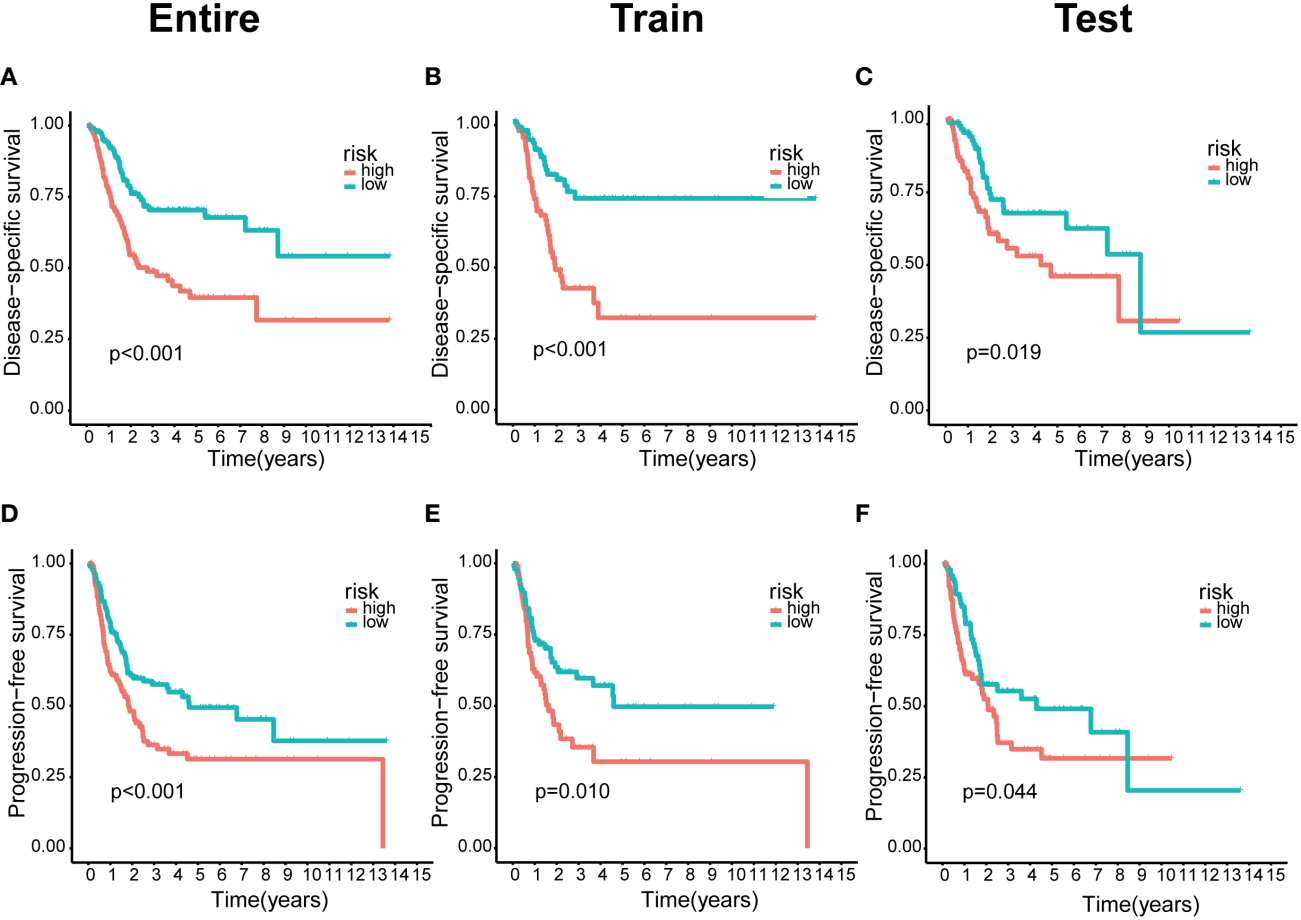
The lower risk group had a better prognosis in the risk model. **(A–C)** Kaplan-Meier curves of disease-specific survival in the entire, train and test sets, respectively, in the high-risk group compared with the low-risk group. **(D–F)** Kaplan–Meier curves of progression-free survival in the entire, train and test sets, respectively, in the high-risk group compared with the low-risk group.

We integrated clinical characteristics from the BCa cohort in TCGA and excluded individuals with a survival duration of fewer than 30 days. A total of 396 patients were randomly allocated to the train and test groups. We identified 22 prognosis-associated DEBMlncRNAs in the train set through univariable Cox analysis. We then performed LASSO Cox regression and multivariate analysis. Ultimately, eight DEBMlncRNAs (AC004034.1, AL662797.1, NR2F1-AS1, SETBP1-DT, AC011503.2, AC093010.2, LINC00649 and LINC02321) were identified to develop a risk model (**Figures 2A, B****)**. The expression levels of eight DEBMlncRNAs differed statistically substantially between the normal and tumor groups from the BCa dataset in TCGA (**Figure 2C–J**). We list the detailed coefficient of the eight lncRNA signatures (**Table 1**). We summarized eight lncRNA-related differential BM genes (cor>0.4) (**Table S1**). The low-risk group had a better prognosis than the high-risk group based on the disease-specific survival and progression-free survival curve (**Figure 3**).

**Table 1:** Eight DEBMlncRNAs with BCa in the TCGA dataset were identified by LASSO analysis.

Gene	HR	Lower 95% CI	Upper 95% CI	Coefficient	*P-value*
AC004034.1	1.572714	1.058455	2.336831	0.633536	0.025017
AL662797.1	0.376602	0.180607	0.785289	0.770694	0.009198
NR2F1-AS1	1.931818	1.217866	3.064313	0.669855	0.005153
SETBP1-DT	2.151293	1.304341	3.548201	0.645962	0.002694
AC011503.2	0.591378	0.413111	0.846571	0.361024	0.004105
AC093010.2	0.733876	0.550232	0.978812	0.477727	0.035230
LINC00649	0.547561	0.343932	0.871752	60.527114	0.011135
LINC02321	1.560638	1.140471	2.135601	0.307869	0.005414

HR, hazard ratio; CI, confidence interval.

### Prognostic features of risk models

**Figure 4:**
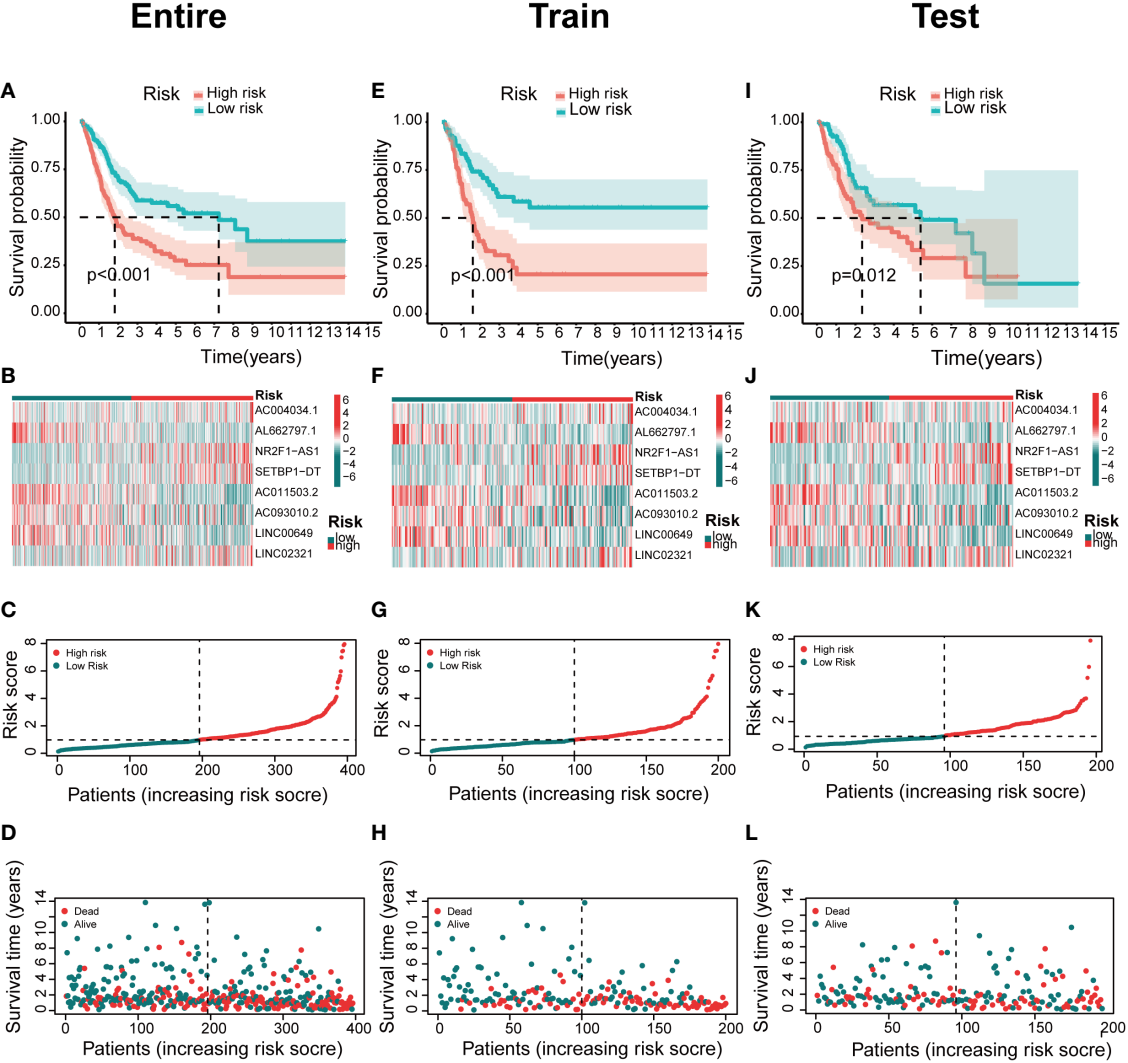
Association between basement membrane-associated lncRNA signatures and prognosis in a risk model. The Kaplan–Meier curves of overall survival, heatmap, risk score, survival time for BCa patients in the **(A–D)** entire set. **(E–H)** train set. **(I–L)** test set.

**Figure 5:**
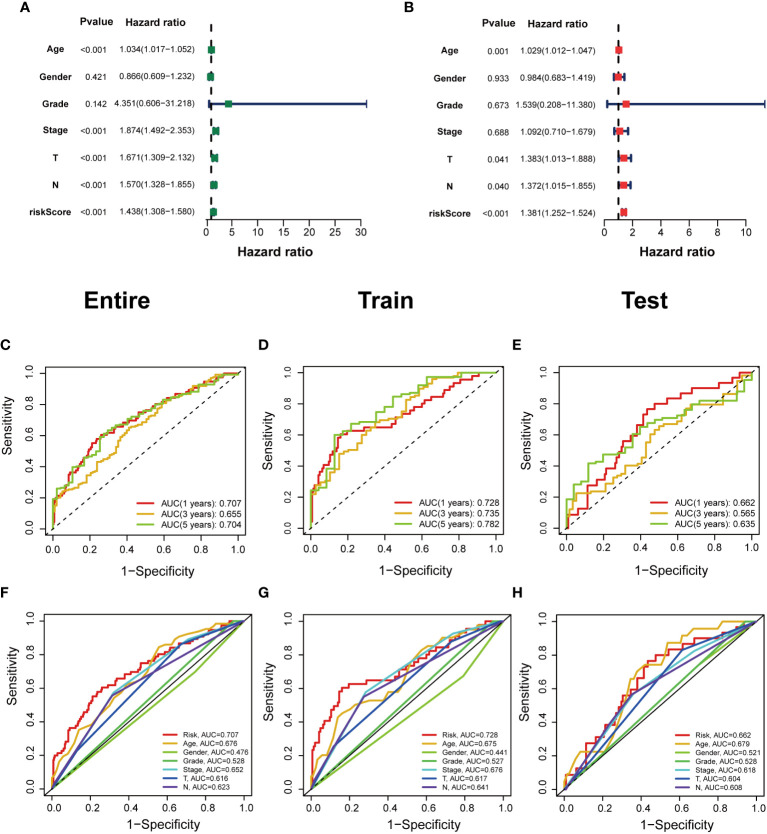
Independent prognostic value of clinical features in risk models. **(A, B)** Univariate Cox regression analysis and multivariate Cox regression analysis of clinical factors and risk scores. **(C–E)** Time-dependent ROC curves predict 1-year, 3-year, and 5-year overall survival for BCa patients in the entire; train; and test sets. **(F–H)** Multivariate time‐dependent ROC curve predicted the AUC for age, gender, grade, stage, T, N, and risk score of the total survival for 1‐year in the entire; train; and test sets.

The predictive value of prognostic features was evaluated in the entire set (n=396), train set (n=200), and test set (n=196). The distribution of risk scores, survival status, survival time, and associated expression criteria for these lncRNAs between low- and high-risk groups was assessed using risk scoring formulas across the entire set, training, and test sets. Based on these findings, The high-risk group had a worse prognosis than the low-risk group (**Figure 4**). The hazard ratio and 95% confidence interval (CI) of the risk score in univariate Cox regression were 1.438 and 1.308-1.580, respectively (*p* < 0.001). The hazard ratio and 95% CI of risk were 1.381 and 1.252-1.524 (p < 0.001), respectively, according to multivariate Cox regression (**Figures 5A, B**). Furthermore, age (1.029 and 1.012–1.047; *p* < 0.001), T (1.383 and 1.013–1.38; *p* < 0.001) and M (1.372 and 1.015–1.855; *p* < 0.001) were also independent prognostic parameters (**Figure 5B**).

The ROC curve was used to assess the sensitivity and specificity of the model to predict prognosis. We further examined the ROC curve results by calculating the area under the ROC curve (AUC). The 1-, 3-, and 5-year AUCs were 0.707, 0.655, and 0.704, respectively, in the entire set; 0.728, 0.735, and 0.782, respectively, in the training set; and 0.662, 0.565, and 0.635, respectively, in the test set (**Figures 5C–E**). The clinical variables and risk score had the strongest predictive capacity according to the risk model’s 1-year ROC curve (**Figures 5F–H**).

### Clinical features and risk scores

We performed clinical analyses based on the clinicopathological characteristics of BCa patients in the TCGA database (**Table 2**). Heatmap revealed the expression of 8 specific DEBMlncRNAs in high- and low-risk groups. In the BCa data from the TCGA database, there is a statistically significant difference in M, T, stage, gender between the two risk subgroups (**Supplementary Figure 1A**). Through further analysis, we found a significant relationship between risk score and clinical characteristics, age, gender, grade, M, N, and T stages, and the findings showed that women and men had higher risk scores in BCa patients. Patients with the M1 stage have higher risk scores than the M0 stage. Stage II and stage III, stage III and stage IV, T2 and T3, *etc.*, had significant statistical differences in risk scores compared to Stage I and T1, respectively. At the same time, there was no statistically significant relationship between risk score and age, grade, or N stage (**Supplementary Figures 1B–H**).

**Table 2:** Clinicopathological parameters of BLCA patients in this research.

Covariates	Type	Total	Test	Train
**Age (years)**	≤65	159 (40.15%)	84 (42.86%)	75 (37.5%)
	>65	237 (59.85%)	112 (57.14%)	125 (62.5%)
**Gender**	FEMALE	104 (26.26%)	57 (29.08%)	47 (23.5%)
	MALE	292 (73.74%)	139 (70.92%)	153 (76.5%)
**Grade**	High Grade	375 (95.42%)	184 (95.34%)	191 (95.5%)
	Low Grade	18 (4.58%)	9 (4.66%)	9 (4.5%)
**Stage**	Stage I-II	126 (31.98%)	69 (35.57%)	57 (28.5%)
	Stage III-IV	268 (68.02%)	125 (64.43%)	143 (71.5%)
**T-stage**	T0-T2	117 (32.14%)	68 (37.57%)	49 (26.77%)
	T3-T4	247 (67.82%)	113 (62.43%)	134 (73.22%)
**M-stage**	M0	189 (94.97%)	89 (95.7%)	100 (94.34%)
	M1	10 (5.03%)	4 (4.3%)	6 (5.66%)
**N-stage**	N0-N1	273 (76.9%)	130 (75.14%)	143 (78.57%)
	N2-N3	82 (23.1%)	43 (22.86%)	39 (21.43%)

BLCA, bladder cancer; T, tumor; N, node; M, metastasis.

### Nomogram and clinical indicators

**Figure 6:**
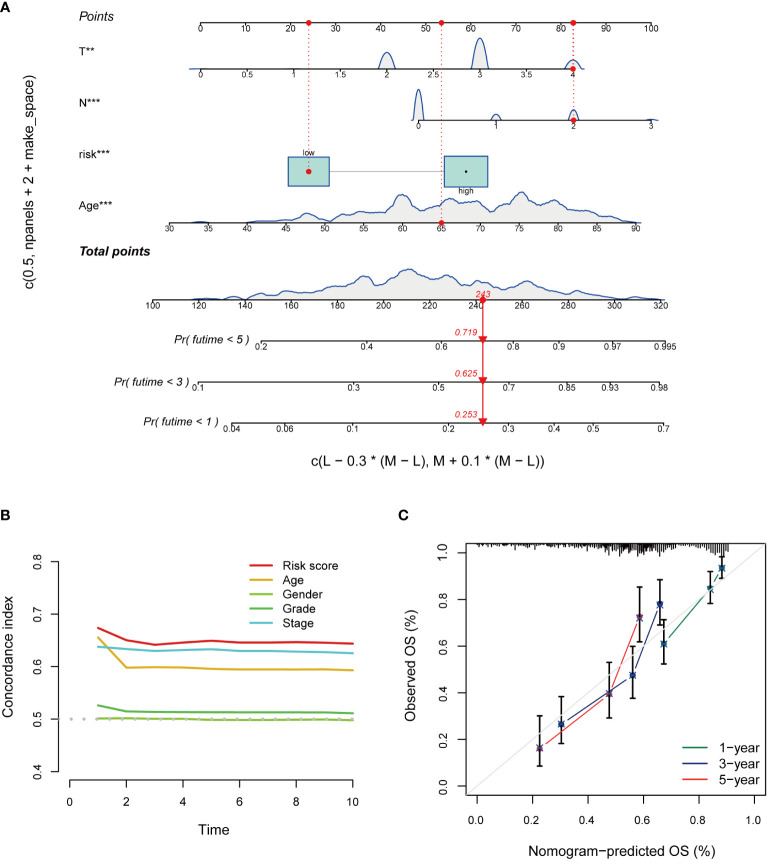
Construction and evaluation of the nomogram of the risk model. **(A)** Nomogram combines risk scores and clinical features to predict 1-, 3-, and 5-year overall survival. **(B)** Concordance index of risk score and clinical characteristics. **(C)** The calibration curves for 1-, 3-, and 5-year overall survival. *P < 0.05, **P < 0.01, ***P < 0.001.

**Figure 7:**
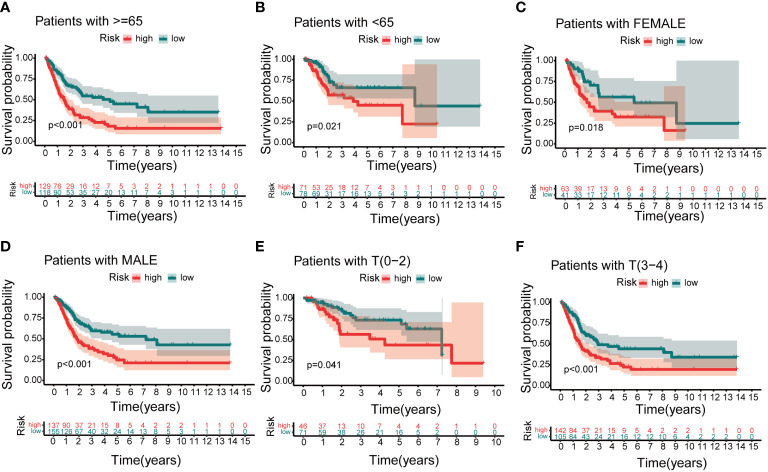
The clinical utility of the risk score. **(A, B)** Age. **(C, D)** Gender. **(E, F)** T.

Based on independent prognostic factors, namely risk score, age, T, N, stage, gender, and grade, we used nomograms to predict 1-, 3-, and 5-year overall survival in BCa patients (**Figure 6A**). We found that risk scores had the highest concordance index, suggesting that risk scores were more accurate than other clinical information in predicting prognostic outcomes (**Figure 6B**). We also used 1-, 3-, and 5-year calibration plots to verify the nomogram’s agreement with the overall survival prediction (**Figure 6C**). Survival curves indicated that patients in the high-risk group with age, gender, T0-2 and T3-4 disease had a poorer prognosis (**Figures 7A–F**).

### Functional and pathway enrichment analysis in risk model

**Figure 8:**
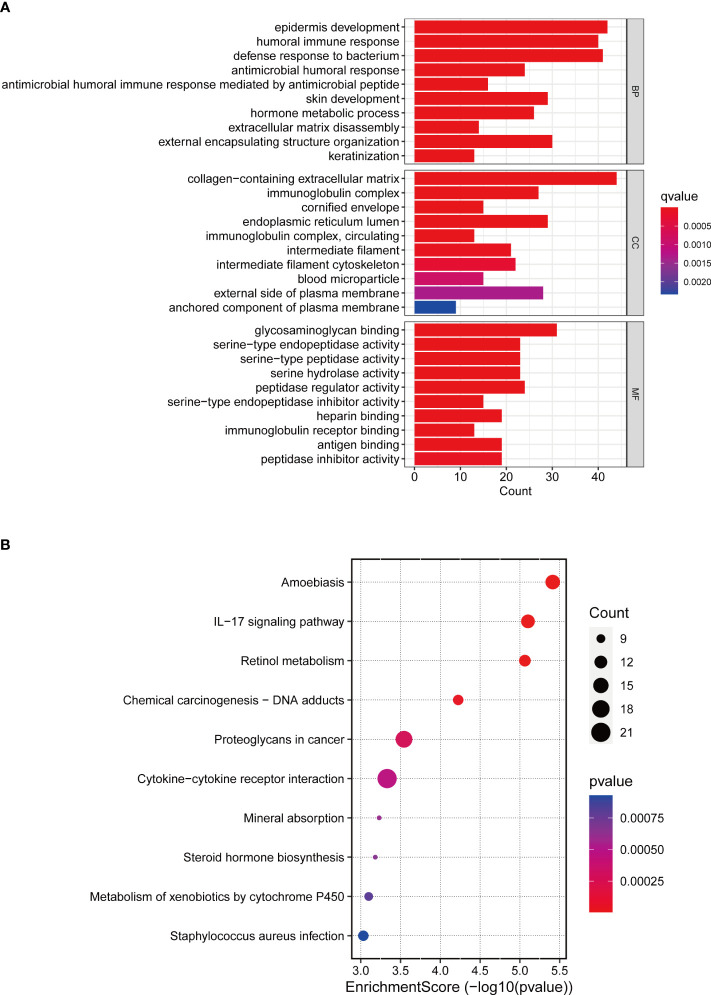
GO and KEGG enrichment analysis. **(A)** GO enrichment analysis in the differentially expressed genes between the low-risk and high-risk groups. **(B)** KEGG pathway analysis in the differentially expressed genes between the low-risk and high-risk groups.

We used GO, KEGG and GSEA to analyze DEGs in low-risk and high-risk groups to better understand the underlying biological processes. In the GO analysis, DEGs were mainly enriched in BCa-related biological processes, including “Epidermis development”, “Humoral immune response”, “Extracellular matrix disassembly”, and “Keratinization” (**Figure 8A**). According to the KEGG analysis, these DEGs were mainly enriched in the “IL−17 signaling pathway”, “Proteoglycans in cancer” (**Figure 8B**). In the GSEA analysis, “Ecm receptor interaction” and “Regulation of actin cytoskeleton” were mainly enriched in the high-risk group. The low-risk group was enriched in “arachidonic acid metabolism”, “fatty acid metabolism”, and “retinol metabolism” (**Supplementary Figure 2**).

### Tumor mutational burden analysis in risk model

**Figure 9:**
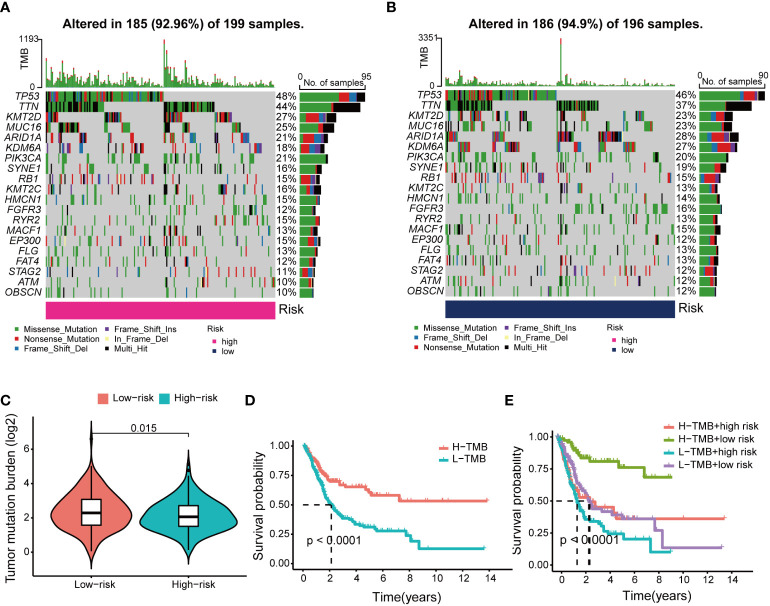
Differences and prognosis in tumor mutational burden (TMB). **(A)** The waterfall plot and heatmap of mutation genes in the high-risk group. **(B)** The waterfall plot and heatmap of mutation genes in the low-risk group. **(C)** Differences in TMB scores across risk groups. **(D)** Kaplan-Meier curves of H-TMB group and L-TMB group. **(E)** Kaplan-Meier curve of H-TMB and L-TMB scores in the different risk groups.

After classifying BCa patients into high TMB (n = 185) and low TMB (n = 186) groups based on the median TMB, the waterfall plot showed that the highest mutated genes between the two risk groups were TP53 and TTN scores (**Figures 9A, B****)**. The mutation frequency of KDM6A was 27% in the low-risk group and 18% in the high-risk group. Further analysis found that patients in the low-risk group had higher TMB scores than those in the high-risk group (**Figure 9C**). In the Kaplan–Meier results, a high TMB increased patient survival compared to a low TMB (**Figure 9D**). Interestingly, various groups of patients with varying TMB scores showed diverse prognoses in this research. In the Kaplan–Meier analysis, patients in the low-risk category with high TMB had a significantly better prognosis than patients in other categories. (**Figure 9E**).

### Immune cell infiltration, immune function analysis and immunotherapy

We assessed differences in immune function between the two groups by calculating the ESTIMATE score, immune score, and stromal score of BCa samples. The results of the study found that patients in the high-risk group had higher scores in all three categories (**Supplementary Figures 3A–C**). By further exploring the distribution of immune cells in BCa patients, we found that the low-risk group was enriched with a high number of B cells naive, plasma cells, T cells follicular helper, T cells regulatory and dendritic cells activated, whereas the high-risk group contains a higher proportion of NK cells resting (**Supplementary Figure 3D**). By analyzing the correlation of immune cell infiltration with risk score, we found that risk score was significantly negatively associated with the function of B cells naive, plasma cells, T cells follicular helper, T cells regulatory and dendritic cells activated and were significantly positively correlated with NK cells resting (**Supplementary Figures 3E–I**).

Our study found 33 immune checkpoints (LAIR1, TNFSF18, TNFSF9, TNFRSF25, TNFRSF9, TNFRSF18, ICOS, CD70, PDCD1, HAVCR2, CD44, BTLA, PDCD1LG2, CTLA4, CD274, LAG3, TNFRSF14, TNFRSF8, CD80, CD48, LGALS9, IDO1, TNFSF4, TNFRSF4, CD27, CD28, NRP1, CD86, CD276, TIGIT, TNFSF14, TNFSF15, and CD160) that were statistically different in the high-risk and low-risk groups (**Supplementary Figure 4A**). Through the analysis of immune function, 12 immune function scores in the high-risk group were higher than those in the low-risk group (**Supplementary Figure 4B**). In addition, tumor immune dysfunction and exclusion (TIDE) scores were significantly higher in the high-risk group (**Supplementary Figure 4C**). We classified BCa patients into four immune subtypes (C1, C2, C3, and C4), with C2 having the highest risk score, followed by C1, and finally C3 and C4, with no statistical difference between C3 and C4 (**Supplementary Figure 4D**).

With the popularity of personalized medicine in recent years, immunosuppressive treatment, such as immune checkpoint inhibitor (ICI) therapy, has also gained much attention. When considering clinical value, the high-risk group was more sensitive to A.443654, A.770041, AICAR, AMG.706, AUY922, AZ628, and AZD.0530 than the low-risk group. The low-risk group was more sensitive to ABT.888, AKT. Inhibitor VIII, ATRA and Axitinib (**Supplementary Figure 5**).

### Identification of eight BM-associated LncRNAs

**Figure 10:**
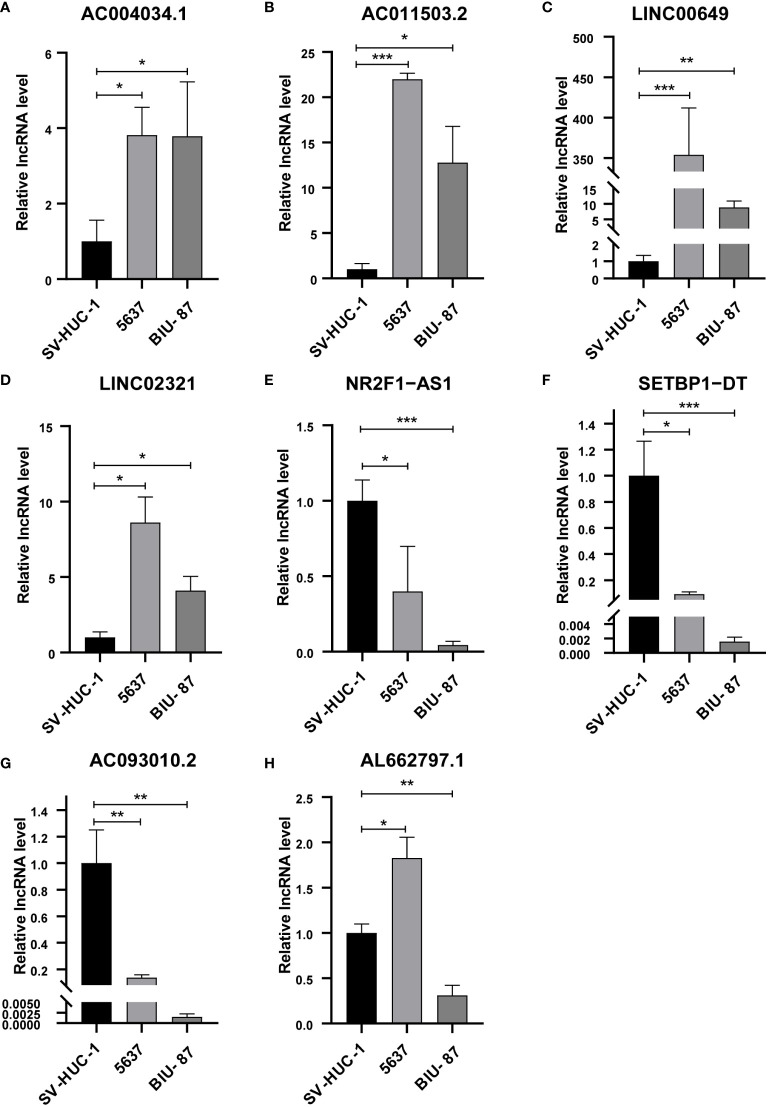
qRT-PCR for detection of lncRNA expression levels in a risk model in BCa cells. **(A)** AC004034.1 **(B)** AC011503.2 **(C)** LINC00649 **(D)** LINC02321 **(E)** NR2F1-AS1 **(F)** SETBP1-DT **(G)** AC093010.2 **(H)** AL662797.1 *P < 0.05, **P < 0.01, ***P < 0.001.

We analyzed the expression levels of eight lncRNAs in BCa and paracancerous tissues in the GSE133624 chip. AC011503.2, LINC00649, and LINC02321 were highly expressed in BCa, NR2F1-AS1, SETBP1-DT, and AC093010.2 were under expressed in BCa. This is consistent with the expression trend of six lncRNAs in BCa and paracancerous tissues in the TCGA database (**Supplementary Figure 6**). We analyzed the expression of eight BM-associated lncRNAs and validated them in normal human bladder epithelial cells and human bladder cancer cells. The study showed that AC004034.1, AC011503.2, LINC00649 and LINC02321 (**Figures 10A–D**) were significantly elevated in human BCa cells, NR2F1-AS1, SETBP1-DT and AC093010.2 (**Figures 10E–G**) were significantly reduced in human bladder cancer cells. The expression of AL662797.1 (**Figure 10H**) was significantly increased in 5637 cells and significantly decreased in BIU-87 cells.

### RNA FISH and CCK8

**Figure 11:**
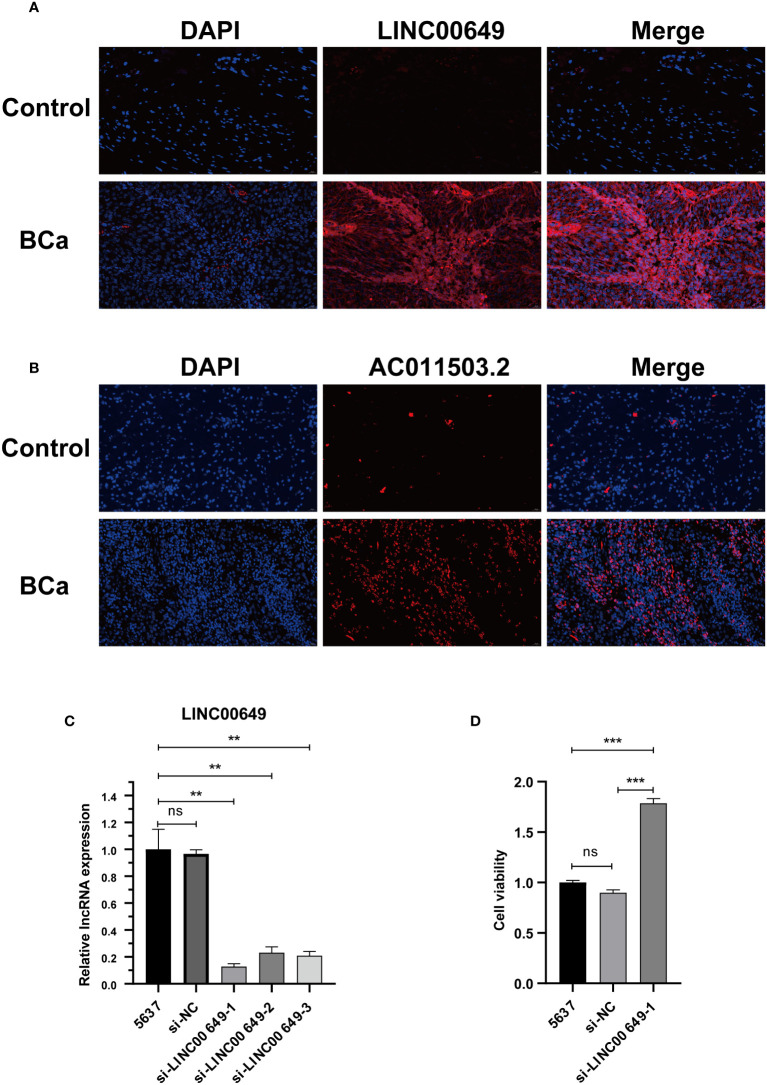
Validate the expression of model lncRNAs in tissues and explore the effect on 5637 cell proliferation. **(A)** RNA-FISH showed that LINC00649 was mainly distributed in the cytoplasm. **(B)** RNA-FISH showed that AC011503.2 was mainly distributed in the nucleus. **(C)** The effects of si-LINC00649 on LINC00649 expression in 5637 cells. **(D)** The effects of knockdown of LINC00649 on the proliferation viability of 5637 cells. **P > 0.01, ***P >0.001, ns, not significant.

We verified and localized the expression of LINC00649 and AC011503.2 in BCa tissue microarray by RNA FISH technology. We found that both LINC00649 and AC011503.2 were highly expressed in BCa tissues, LINC00649 was mainly distributed in the cytoplasm, and AC011503.2 was distributed primarily in the nucleus (**Figures 11A, B****)**. We used si-LINC00649 to knock down the expression of LINC00649 in 5637 cells, and si-LINC00649-1 had the best knockdown effect (**Figure 11C**). Through CCK8 detection, we found that knockdown of LINC00649 could promote the proliferation of 5637 cells (**Figure 11D**).

## Discussion

BCa is one of the most common invasive tumors in urology. At present, the main clinical treatment methods are surgical resection and chemotherapy. However, in recent years, the prognosis of BCa patients has not been effectively improved due to tumor metastasis and recurrence (24). As the main barrier of carcinoma *in situ* and invasive tumors, the BM ensures the integrity of its structure and function, can control the metastasis of cancer cells, delay the progression of cancer, and improve the prognosis of patients. The BM is mainly composed of a fibrin network consisting of collagen IV and laminin. There is substantial evidence that tumor invasion is associated with collagen IV and laminin (25). As in invasive BCa, the expression level of serum laminin P1 is proportional to disease progression (26). Loss of laminin 5 expression in immunohistochemistry is strongly associated with increased BCa mortality (27). Highly aggressive tumors secrete laminin and collagen IV, and overexpression of these two proteins promotes tumor growth and angiogenesis (28). The usefulness of BM detection for the classified staging of BCa has also reached widespread recognition (25). These all reflect the importance of the BM for the clinical progression of BCa. However, the BM’s specific components and regulatory mechanisms are still unclear. The latest research screened BM-related proteins through bioinformatics technology and a large amount of disease genome information and finally found 222 protein expression genes closely related to BM (29). Although many types of lncRNAs are related to tumor prognosis in previous studies, such as immune-related lncRNAs, epithelial-mesenchymal transition-related lncRNA, redox-related lncRNA, etc (30–32). However, there are currently few approaches for predicting BM-related lncRNAs in BCa patients.

In this study, we developed a BM-related lncRNA prognostic model based on the genetic information of the BM-BASE database. In our study, 8 BM-related lncRNAs, including AC004034.1, AL662797.1, NR2F1-AS1, SETBP1-DT, AC011503.2, AC093010.2, LINC00649and LINC02321, were selected by LASSO regression to construct a risk model based on DEBMlncRNAs. We used the dataset GSE133624 for external validation of 8 lncRNAs, of which the expression results of 6 lncRNAs(AC011503.2, LINC00649, LINC02321, NR2F1-AS1, SETBP1-DT, and AC093010.2) were consistent with the results in the TCGA database. Currently, we have verified the expression of eight lncRNAs in SV-HUC-1, 5637, and BIU-87. SV-HUC-1 cells served as controls; AC004034.1, AC011503.2, LINC00649, and LINC02321 were highly expressed in 5637, BIU-87 cells, NR2F1-AS1, SETBP1-DT, and AC093010.2 in 5637, BIU-87 cells Moderate to low expression. These are consistent with the expected results. However, AL662797.1 is highly expressed in 5637 cells and low in BIU-87 cells, and the mechanism needs further exploration. In a human bladder cancer chip, we examined the expression and cellular localization of LINC00649 and AC011503.2. We found that LINC00649 and AC011503.2 were highly expressed in bladder cancer tissues, LINC00649 was mainly distributed in the cytoplasm, and AC011503.2 was distributed primarily in the nucleus. Furthermore, we knocked down the expression of LINC00649 in 5637 cells and found that it promoted the proliferation of 5637 cells. However, the specific mechanism needs further research and verification. Previous studies found that NR2F1-AS1 upregulates the expression level of ST8SIA1 by recruiting SPI1, thereby promoting proliferation and metastasis of BCa cells (33). LINC00649 has verified its tumor-promoting effect in gastric cancer, colorectal cancer, BCa, etc (34–36). LINC02321 is upregulated in BCa tissues and UMUC3 cells (37). AC004034.1, AL662797.1, SETBP1-DT, AC011503.2 and AC093010.2 have been less studied and will be explored further. We focused on DMlncRNAs and pathways by investigating the correlation between gene expression and gene mutation using clinical data from normal and BCa samples reported by TCGA to determine whether DMlncRNAs are potential targets for BCa therapy. Patients with higher risk scores were more likely to have adverse outcomes than patients with lower risk scores. TMB analysis showed significant differences in the mutated genes KDM6A, ARID1A and TTN and prognosis among different groups, and their gene functions need to be further studied. From the immune microenvironment, we can see that patients in the high-risk group have more resting NK cell infiltration. In addition, patients in the low-risk group had higher proportions of naive B cells, plasma cells, T cell follicular helper cells, regulatory T cells, and activated dendritic cells. This may be the reason for the poorer prognosis in the high-risk group, and further studies are needed to prove our results. Interestingly, low-risk and high-risk group patients were sensitive to multiple chemotherapeutic agents. Immune checkpoint analysis revealed that 33 genes based on this risk score were significantly different.

Several immune checkpoint inhibitors (ICIs) are currently approved as first-line therapy in patients who are not suitable for cisplatin or second-line therapy in metastatic urothelial carcinoma (MUC) of the bladder. About 30% of MUC patients will respond to ICIs immunotherapy. Immunosuppressants currently developed for PD-1 and PD-L1 show better prognostic effects than chemotherapy in the second-line treatment of MUC (38). ICIs targeting CTLA4, such as ipilimumab and tremelimumab, have also proven therapeutic effects in treating advanced BCa (39). Extracellular matrix disassembly and remodeling can affect tumor invasion by altering the tumor microenvironment (40). Keratinization is also associated with tumor metastasis, with transformed keratinocytes invading the dermis through the BM leading to aggressive cutaneous squamous cell carcinomas with substantial metastatic potential (41). IL-17 signaling pathway is also a classic cancer-related signaling pathway, which has been extensively studied in breast cancer, colorectal cancer, and squamous cell carcinoma (42–44).

Proteoglycans in BM are aberrantly expressed or dysfunctional upon stimulation by cancer cells and affect cancer and angiogenesis, and are critical to the evolution of the tumor microenvironment (45). Tumor mutational burden is an important biological marker indicating tumor mutation status and is considered as an effective method to discover potential tumor immune regulatory pathways (46, 47). In this study the prognostic survival rate of H-TMB is higher than that of L-TMB, indicating that the prognosis of L-TMB patients in the high-risk group is the worst and requires earlier combined treatment and targeted therapy. In the early stages of tumors, immune cells can interact with cancer cells through the BM barrier to control cancer progression (48). In our study, patients in the high-risk group tend to have higher tumor stages, which may be related to changes in the structure and function of the basement membrane. Still, more researches are needed to prove our results further.

In recent years, with the maturity of sequencing technology, big data mining has gradually emerged and promoted the process of personalized medicine. Although some BM-related proteins have been studied in BCa in the past, the core BM-related proteins have not been summarized and modeled. The BM-related protein expression genes were downloaded from the BASE database, and these genes were used for further research and modeling in BCa. Currently, risk scoring models are mostly constructed using the LASSO regression method. The ROC curve can also indicate that the risk model has higher sensitivity and specificity in predicting prognosis than other clinical indicators. At the same time, the correlation analysis between risk scores and clinical index showed statistical differences in immune scores in terms of T stage, M stage and gender. This result implies that high-risk scores are associated with aggressive BCa as well as metastatic BCa, and are consistent with prognostic outcomes in prior-risk subgroups. Immune checkpoint inhibitors are a promising cancer therapy that blocks key molecules in the development of cancer to demonstrate anticancer efficacy, especially in patients with advanced cancer and those who cannot afford chemotherapy (49, 50).

In conclusion, our study provides a basis for the study of basement membranes in BCa. First, we developed prognostic risk models for 8 DEBMlncRNAs through public databases, found that the risk models could accurately predict the prognosis of patients, and the risk score was also closely related to tumor invasion and metastasis and verified the expression of 8 DEBMlncRNAs in BCa cells. Second, we confirmed the expression of LINC00649 and AC011503.2 in human bladder cancer tissue microarray by RNA-FISH. Third, we knocked down the expression of LINC00649 in 5637 cells with si-LINC00649 and demonstrated that the knockdown of LINC00649 could promote the proliferation of 5637 cells.

## Conclusions

In conclusion, we identified eight BM-associated lncRNAs for predictive signature and risk modeling. This risk model can predict the prognosis and immune status of BCa patients, classify the degree of tumor infiltration and metastasis, and thus provide a favorable early personalized treatment option for BCa patients.

## Data availability statement

Publicly available datasets were analyzed in this study. This data can be found here: The data are available at the TCGA (https://tcga-data.nci.nih.gov/tcga/) and BM-BASE database (https://bmbase.manchester.ac.uk).

## Author contributions

LF and PL designed the study, and JY, LL, WZ, and LF analyzed the data. LF and LL drafted the manuscript. XW, PL and MP revised the original manuscript. All the authors approved the final manuscript.

## Funding

This study was supported by the National Natural Science Foundation of China (No.81770688), Wuhan Medical Research Project (WX21B04), Hubei Leading Talent Program in Medicine, and Wuhan Application Foundation and Frontier Project (No. 2020020601012209).

## Acknowledgments

We express our sincere gratitude for using data from TCGA and the BM-BASE database.

## Conflict of interest

The authors declare that the research was conducted in the absence of any commercial or financial relationships that could be construed as a potential conflict of interest.

## Publisher’s note

All claims expressed in this article are solely those of the authors and do not necessarily represent those of their affiliated organizations, or those of the publisher, the editors and the reviewers. Any product that may be evaluated in this article, or claim that may be made by its manufacturer, is not guaranteed or endorsed by the publisher.
