# Supplementary material for: Prognostic significance and identification of basement membrane-associated lncRNA in bladder cancer
Source: Front Oncol. 2022 Oct 10;12:994703. doi: 10.3389/fonc.2022.994703 (PMC9590283; doi:10.3389/fonc.2022.994703)
Supplement: Supplementary file 1 [file DataSheet_1.docx]

Supplementary Material

Table S1. Correlation of 8 lncRNAs with differential BM genes.

| **mRNA** | **lncRNA** | **Cor** | ***P-value*** |
| --- | --- | --- | --- |
| FGF9 | AC004034.1 | 0.614524219 | 2.38E-44 |
| GPC2 | AL662797.1 | 0.42860384 | 6.29E-20 |
| LAMA4 | NR2F1-AS1 | 0.475665835 | 9.21E-25 |
| SMOC2 | SETBP1-DT | 0.417674773 | 6.57E-19 |
| SPARCL1 | SETBP1-DT | 0.409821443 | 3.36E-18 |
| ADAMTS20 | AC011503.2 | 0.422239807 | 2.49E-19 |
| ADAMTS8 | AC093010.2 | 0.439559535 | 5.49E-21 |
| ADAMTS9 | AC093010.2 | 0.402329996 | 1.54E-17 |
| FBLN5 | AC093010.2 | 0.52885041 | 3.30E-31 |
| HMCN2 | AC093010.2 | 0.432106175 | 2.91E-20 |
| LAMA2 | AC093010.2 | 0.412029967 | 2.13E-18 |
| OGN | AC093010.2 | 0.475024652 | 1.08E-24 |
| PODN | AC093010.2 | 0.485465761 | 7.24E-26 |
| RECK | AC093010.2 | 0.471850096 | 2.43E-24 |
| SLIT3 | AC093010.2 | 0.425813076 | 1.15E-19 |
| SMOC2 | AC093010.2 | 0.507943083 | 1.54E-28 |
| SPARCL1 | AC093010.2 | 0.571346203 | 3.03E-37 |
| DDR2 | AC093010.2 | 0.552283294 | 1.98E-34 |
| ITGA7 | AC093010.2 | 0.510169375 | 8.17E-29 |
| ITGA8 | AC093010.2 | 0.456399609 | 1.08E-22 |
| ADAM10 | LINC00649 | 0.417165887 | 7.31E-19 |
| FBN2 | LINC02321 | 0.414495101 | 1.28E-18 |

Cor, correlation.


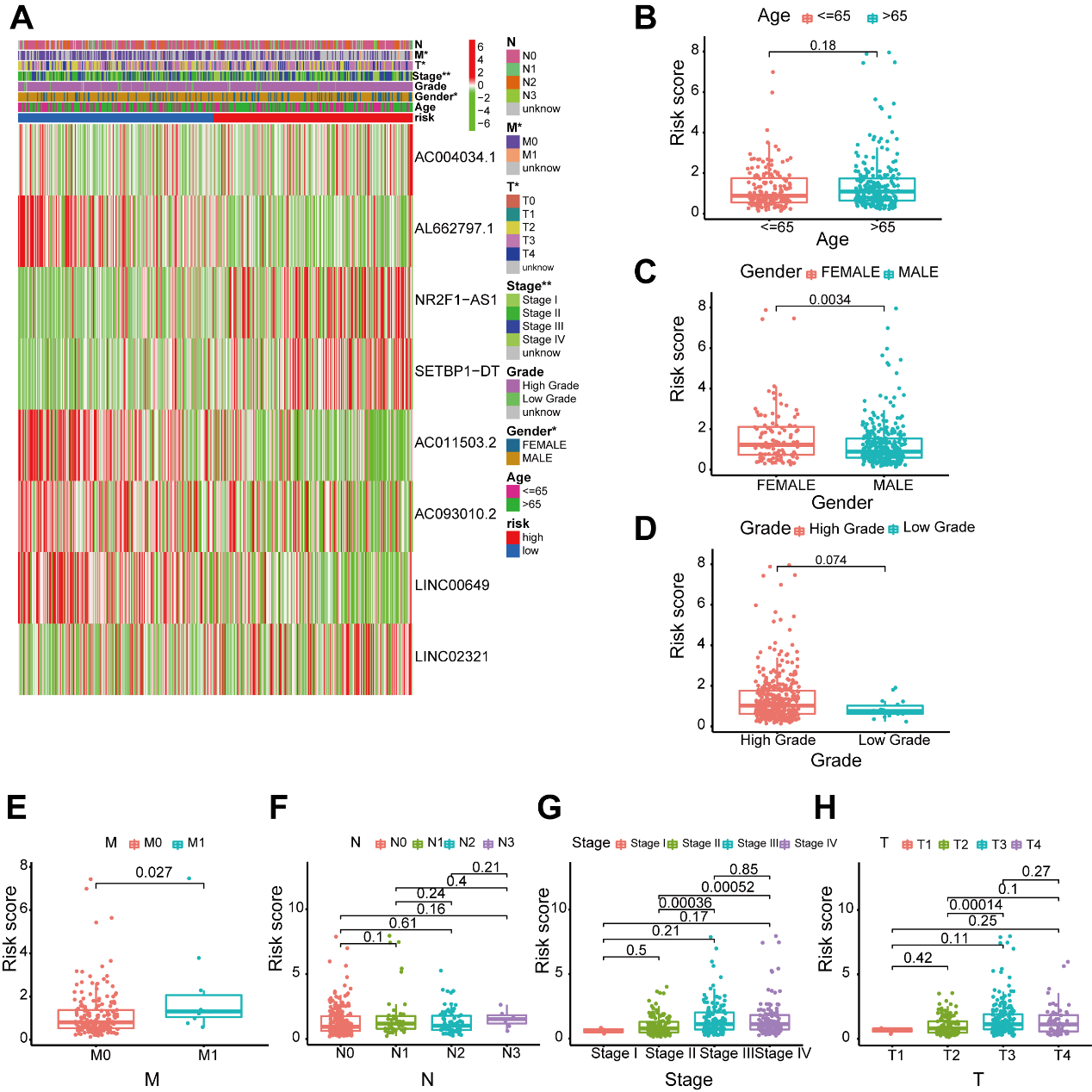


**Supplementary Figure 1.** The correlations of risk score with clinical features and Clinical Heatmap. **(A)** Heatmap of clinical features. **(B)** Age. **(C)** Gender. **(D)** Grade. **(E)** M. **(F)** N. **(G)**Stage. **(H)** T. **P* < 0.05, ***P* < 0.01, ****P* < 0.001.


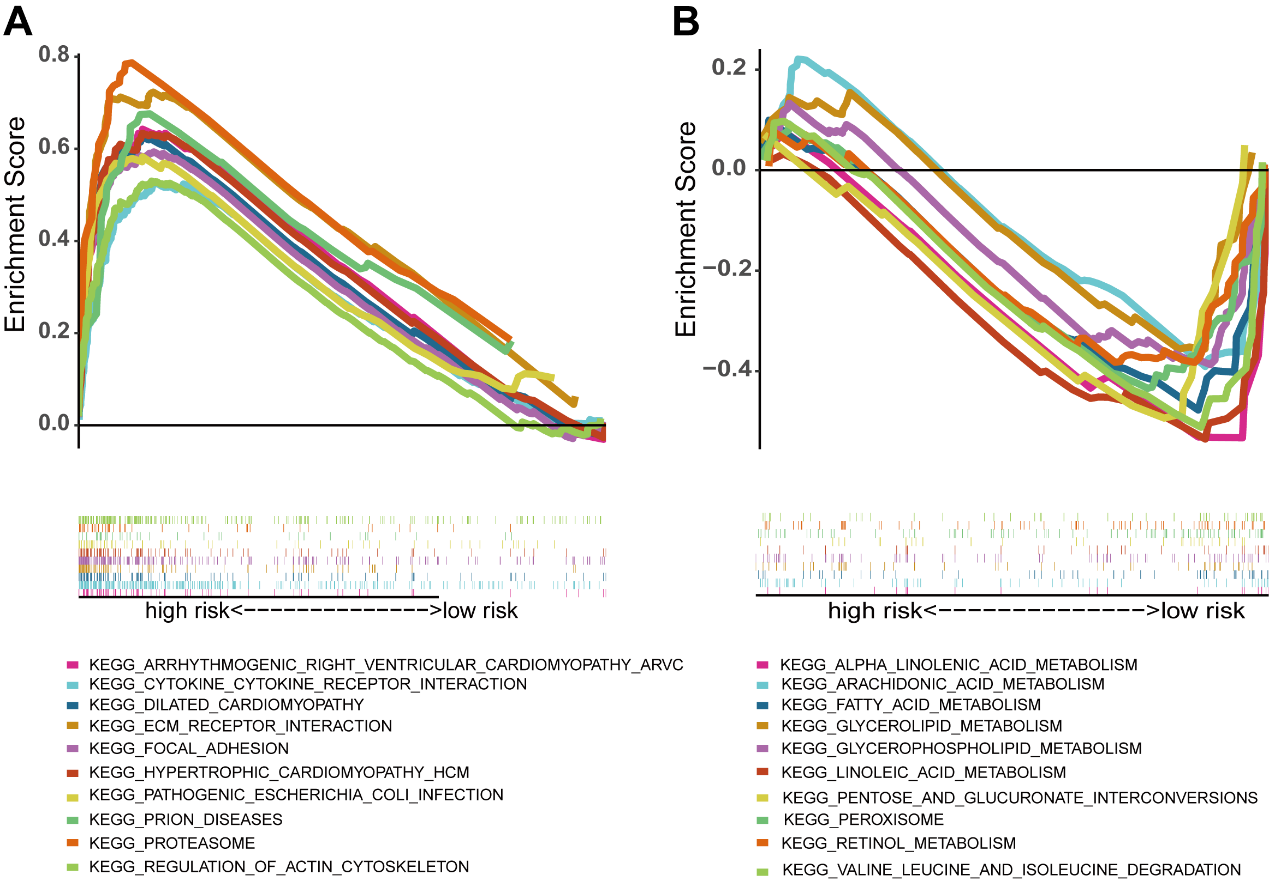


**Supplementary Figure 2.** Gene set enrichment analysis. **(A)** KEGG enrichment pathways in the high-risk group. **(B)** KEGG enrichment pathways in the low-risk group.


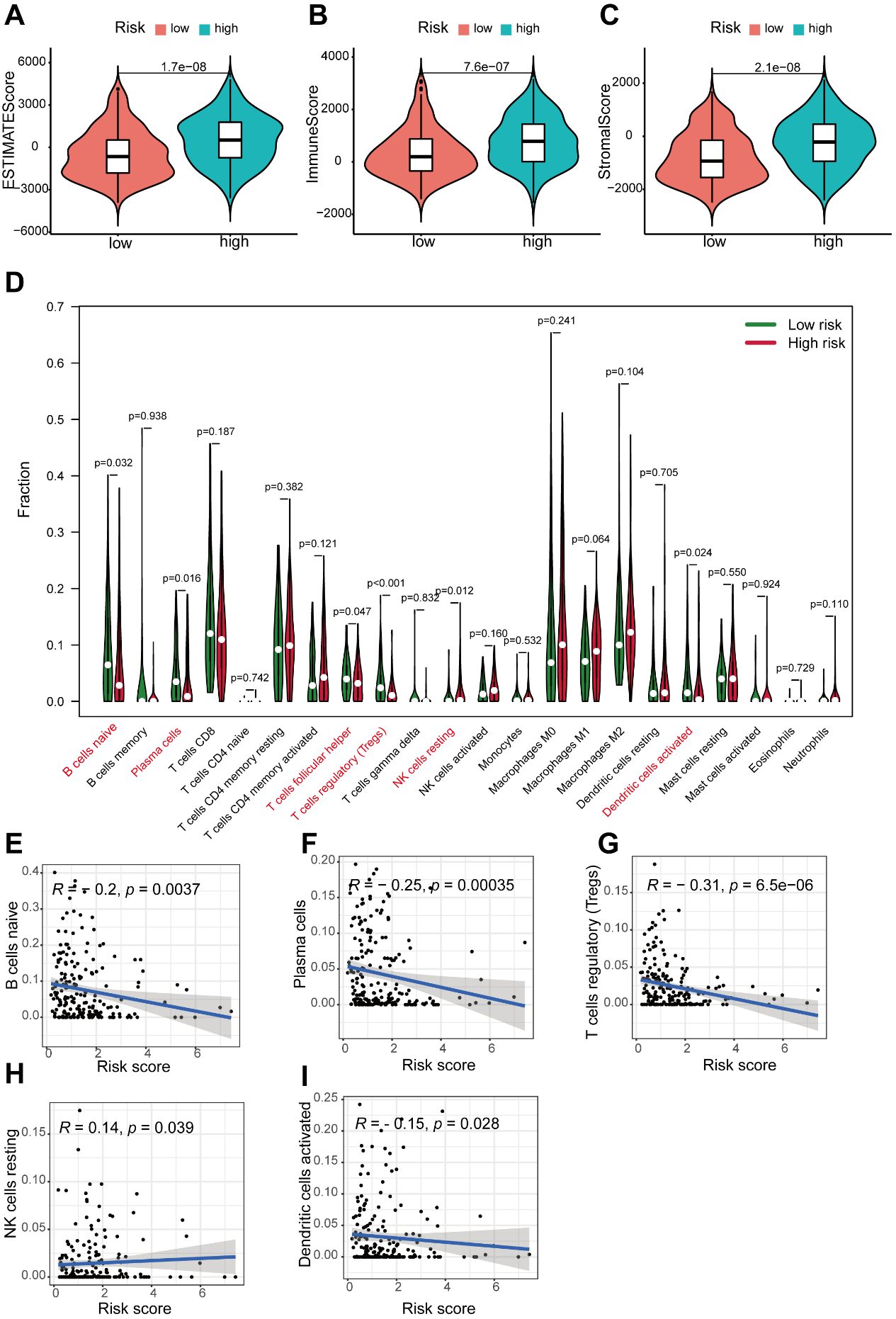


**Supplementary Figure 3.** Differences and correlations between tumor-infiltrating immune cells and risk scores. **(A-C)** Tumor microenvironment scores in high-risk and low-risk groups. **(D)** Different immune cell infiltration in low-risk and high-risk groups. **(E-I)** The correlation between risk score and immune cells.


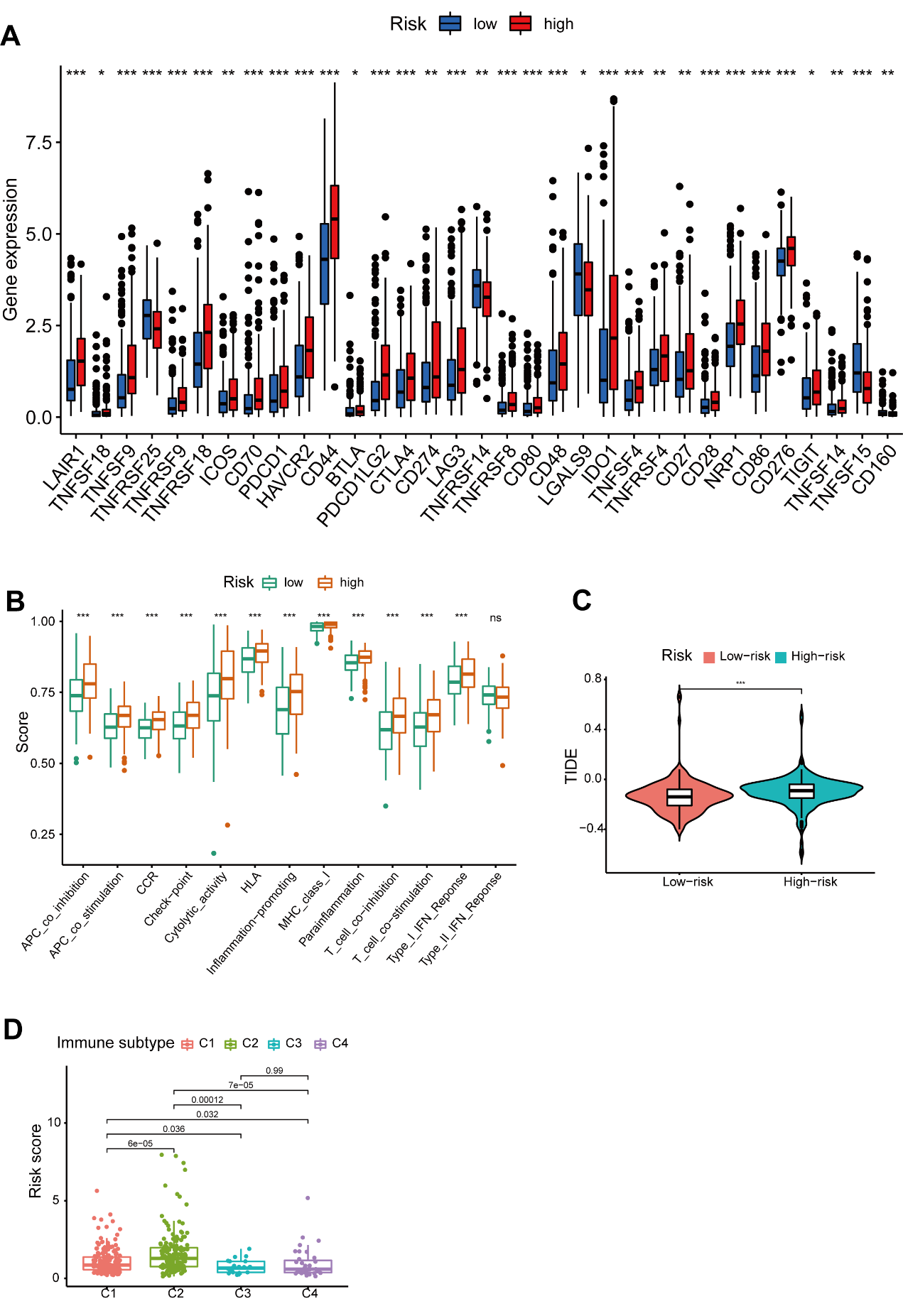


**Supplementary Figure 4.** Correlation of immune checkpoints, immune function and immunophenotyping with risk scores. **(A)** The differential expression of the 33 immune checkpoints in the risk groups. **(B)** Scores of 13 immune-related functions in high-risk versus low-risk groups. **(C)** The boxplot representation of TIDE scores in the high-risk group and low-risk group. **(D)** The correlations of risk score with immune subtype. **P* < 0.05, ***P* < 0.01, ****P* < 0.001.


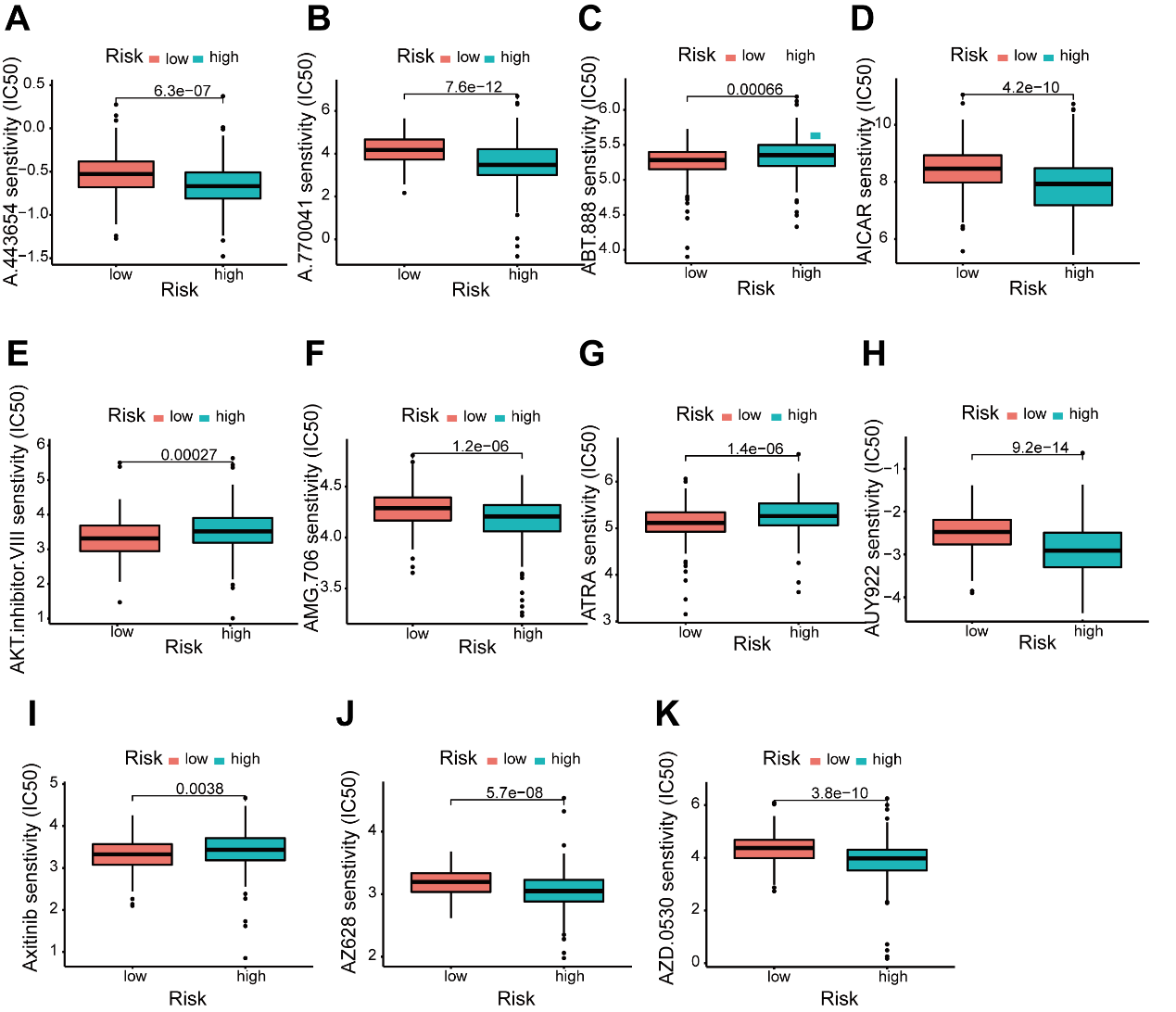


**Supplementary Figure 5.** Eleven immunotherapeutic drugs solely showed a significant IC50 difference in risk groups. **(A)** A.443654 **(B)** A.770041 **(C)** ABT.888 **(D)** AICAR **(E)** AKT.inhibitor.VIII **(F)** AMG.706 **(G)** ATRA (H) AUY922 **(I)** Axitinib **(J)** AZ628 **(K)** AZD.0530


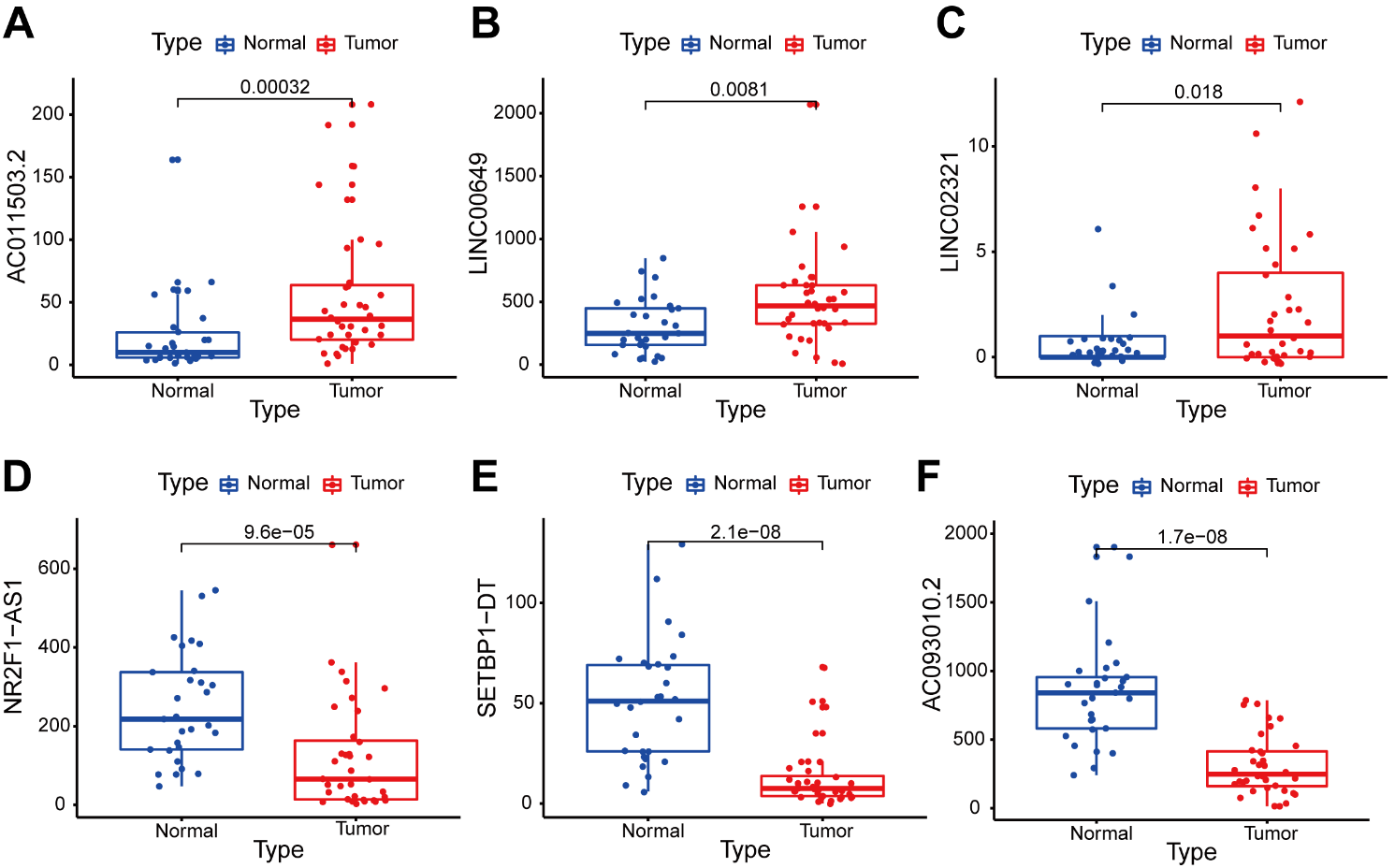


**Supplementary Figure 6.** Validation of the expression of 6 lncRNAs in bladder cancer and paracancerous tissues in GSE133624. **(A)** AC011503.2 **(B)** LINC00649 **(C)** LINC02321 **(D)** NR2F1-AS1 **(E)** SETBP1-DT **(F)** AC093010.2
